# Blunt Chest Trauma in Mice after Cigarette Smoke-Exposure: Effects of Mechanical Ventilation with 100 % O_2_

**DOI:** 10.1371/journal.pone.0132810

**Published:** 2015-07-30

**Authors:** Katja Wagner, Michael Gröger, Oscar McCook, Angelika Scheuerle, Pierre Asfar, Bettina Stahl, Markus Huber-Lang, Anita Ignatius, Birgit Jung, Matthias Duechs, Peter Möller, Michael Georgieff, Enrico Calzia, Peter Radermacher, Florian Wagner

**Affiliations:** 1 Institut für Anästhesiologische Pathophysiologie und Verfahrensentwicklung, Ulm, Germany; 2 Klinik für Anästhesiologie, Universitätsklinikum, Ulm, Germany; 3 Institut für Pathologie, Universitätsklinikum, Ulm, Germany; 4 Laboratoire HIFIH, UPRES EA 3859, PRES l’UNAM, IFR 132, CNRS UMR 6214, INSERM U1083, Université Angers, Département de Réanimation Médicale et de Médecine Hyperbare, Centre Hospitalier Universitaire, Angers, France; 5 Klinik für Unfall-, Hand-, Plastische und Wiederherstellungschirurgie, Universitätsklinikum, Ulm, Germany; 6 Institut für Unfallchirurgische Forschung und Biomechanik, Universitätsklinikum, Ulm, Germany; 7 Abteilung Respiratory Diseases Research, Boehringer Ingelheim Pharma GmbH & Co. KG, Biberach/Riss, Germany; Klinikum rechts der Isar - Technical University Munich - TUM, GERMANY

## Abstract

Cigarette smoking (CS) aggravates post-traumatic acute lung injury and increases ventilator-induced lung injury due to more severe tissue inflammation and apoptosis. Hyper-inflammation after chest trauma is due to the physical damage, the drop in alveolar PO_2_, and the consecutive hypoxemia and tissue hypoxia. Therefore, we tested the hypotheses that 1) CS exposure prior to blunt chest trauma causes more severe post-traumatic inflammation and thereby aggravates lung injury, and that 2) hyperoxia may attenuate this effect. Immediately after blast wave-induced blunt chest trauma, mice (n=32) with or without 3-4 weeks of CS exposure underwent 4 hours of pressure-controlled, thoraco-pulmonary compliance-titrated, lung-protective mechanical ventilation with air or 100 % O_2_. Hemodynamics, lung mechanics, gas exchange, and acid-base status were measured together with blood and tissue cytokine and chemokine concentrations, heme oxygenase-1 (HO-1), activated caspase-3, and hypoxia-inducible factor 1-α (HIF-1α) expression, nuclear factor-κB (NF-κB) activation, nitrotyrosine formation, purinergic receptor 2X_4_ (P2XR_4_) and 2X_7_ (P2XR_7_) expression, and histological scoring. CS exposure prior to chest trauma lead to higher pulmonary compliance and lower PaO_2_ and Horovitz-index, associated with increased tissue IL-18 and blood MCP-1 concentrations, a 2-4-fold higher inflammatory cell infiltration, and more pronounced alveolar membrane thickening. This effect coincided with increased activated caspase-3, nitrotyrosine, P2XR_4_, and P2XR_7_ expression, NF-κB activation, and reduced HIF-1α expression. Hyperoxia did not further affect lung mechanics, gas exchange, pulmonary and systemic cytokine and chemokine concentrations, or histological scoring, except for some patchy alveolar edema in CS exposed mice. However, hyperoxia attenuated tissue HIF-1α, nitrotyrosine, P2XR_7_, and P2XR_4_ expression, while it increased HO-1 formation in CS exposed mice. Overall, CS exposure aggravated post-traumatic inflammation, nitrosative stress and thereby organ dysfunction and injury; short-term, lung-protective, hyperoxic mechanical ventilation have no major beneficial effect despite attenuation of nitrosative stress, possibly due to compensation of by regional alveolar hypoxia and/or consecutive hypoxemia, resulting in down-regulation of HIF-1α expression.

## Introduction

Blunt chest trauma is frequently associated with poly-trauma, and independently contributes to mortality if acute lung injury (ALI) develops [1]. Epidemiological data demonstrate that active or passive cigarette smoking (CS) is associated with the development of ALI after blunt trauma [2], and that active cigarette smoking increases the susceptibility to develop Acute Respiratory Distress Syndrome (ARDS) despite younger age and better overall general health status [3]. Scarce data, however, are only available in experimental animals, and the results are conflicting: in mechanically ventilated rats, pre-challenge CS exposure aggravated tissue inflammation and apoptosis, but had only marginal effects in spontaneously breathing animals [4]. Data on lung mechanics, gas exchange or histological changes were not reported in that study. Moreover, CS exposure even suppressed the pro-inflammatory responses of alveolar macrophages during halothane and isoflurane anaesthesia [5].

Lung contusion due to blunt chest trauma induced both pulmonary and systemic hyper-inflammation, oxidative stress, and enhanced apoptosis [6–8]. The pulmonary and systemic hyper-inflammatory response is due to the physical damage *per se*, the trauma-related drop in alveolar O_2_ tension [6], and the consecutive hypoxemia and tissue hypoxia [9]. Chronic obstructive pulmonary disease (COPD) is also associated with pulmonary and systemic inflammation, oxidative and nitrosative stress, and apoptosis [10,11], at least in part as a result of alveolar hypoxia and hypoxemia [12–15]. Finally, in mice, CS exposure-induced COPD [16,17] lead to a similar degree of pulmonary inflammation [18] as that induced by blunt chest trauma in otherwise healthy littermates [8].

It is well-established that long-term hyperoxia causes ALI characterised by oxidative stress and enhanced cell death [19]. Scarce data, however, are only available on the interaction between CS exposure and hyperoxia: *pre*-natal exposure to the CS component benzo[*a*]pyrene potentiated immediate *post*-natal hyperoxic lung injury [20], and long-term (over five days) *post*-natal exposure to hyperoxia had an additive effect on the histological lung damage and organ dysfunction in CS-induced COPD during adulthood [21]. However, in various animal models resulting from haemorrhage [22–25], ischemia/reperfusion injury [26,27] and poly-microbial sepsis [28–30] short-term ventilation with 100% O_2_ resulted in attenuated inflammation and reduced apoptosis [26,27,29–32], and ultimately improved organ function and survival. Therefore, in anesthetized, resuscitated, and mechanically ventilated mice we tested the hypotheses that 1) CS exposure prior to blunt chest trauma aggravates the post-traumatic pulmonary and systemic inflammatory response and thereby organ dysfunction and injury, and that 2) short-term hyperoxia may attenuate this effect.

## Materials and Methods

The study protocol was approved by the University Animal Care Committee and the federal authorities for animal research of the Regierungspräsidium Tübingen, Baden- Württemberg, Germany (protocol no. 1046). The experiments were performed in accordance with the National Institutes of Health Guidelines on the Use of Laboratory Animals. A total of n = 36 C57BL/6J mice of either gender at an age of 10–16 weeks and weighing 23 ± 2 g were obtained from Charles River (Kisslegg, Germany), housed in isolated, ventilated cages under a 12-hours light-dark cycle, and received food and water *ad libitum*. Four of these mice did not undergo CS exposure, anesthesia, chest trauma, and surgery, and served as controls for immunoblotting and electrophoretic mobility shifts (EMSA).

### Cigarette smoke-induced pulmonary inflammation

In order to address the 1^st^ hypothesis, 16 mice were exposed to cigarette smoke (“CS”) over 3–4 weeks for 5 days per week in an exposure box as described previously [18]. Thereafter, 1 week was allowed as a recovery period prior to the blast experiment. This approach was chosen to avoid any acute stress effect induced by the CS exposure procedure *per se*. Mice received 4 cigarettes (Roth-Händle without filters, tar 10 mg, nicotine 1.0 mg, carbon monoxide 6 mg, Badische Tabakmanufaktur Roth-Händle^®^, Lahr, Germany) on day 1, 6 ones day 2, and 8 ones for the following days of the exposure period lasting for 15 min for each cigarette, which was followed by 8-minutes with fresh air (15 L·min^-1^), and an additional 24-minutes break after each second cigarette. A semi-automatic cigarette lighter and smoke generator with an electronic timer was used to control the exposure (Boehringer Ingelheim Pharma GmbH & Co. KG, Biberach, Germany). Particle concentration was monitored by a real time ambient particle monitor (MicroDust Pro, Casella, Amherst, NH, USA). In pilot experiments, this CS-exposure procedure had not caused any effect on the behavior, body weight, or respiratory pattern. Control animals (“Non-CS”, n = 16) were exposed to room air.

### Anaesthesia, blast wave, surgery, and experimental protocol

Mice were anesthetized with a mixture of 2.5% sevoflurane (Sevorane, Abbott, Wiesbaden, Germany) in 50% O_2_ and N_2_ and buprenorphine i.p. (1 μg·g^-1^). Blunt chest trauma was induced by a single blast wave centred on the thorax as described previously [8]. Briefly, compressed air rapidly ruptures a Mylar polyester film (Du Pont de Nemur, Bad Homburg, Germany), which releases a reproducible single blast wave toward the animal’s mid-sternal chest, and thus induces a reproducible contusion of the lungs without remote organ damage. Immediately after trauma, animals received i.p. ketamine (85 μg·g^-1^), midazolam (0.9 μg·g^-1^), and fentanyl (0.18 μg·g^-1^), and were placed on a procedure bench equipped with a closed-loop system to control body temperature [8,33,34]. An incision was made in the anterior neck to expose trachea, right internal jugular vein, and right carotid artery. The trachea was intubated, and the lungs were mechanically ventilated with a pressure-controlled, lung-protective ventilation strategy using a specially designed small animal ventilator (FlexiVent, Scireq, Montreal, Canada). After a lung recruitment manoeuver consisting of an inspiratory hold at 18 cm H_2_O over 5 seconds, the initial respirator settings were: tidal volume 8 μL·g^-1^, respiratory rate 150 breaths·min^-1^, inspiratory/expiratory time ratio 1:2, PEEP 5 cm H_2_O. Recruitment manoeuvers were repeated hourly, because this approach allowed maintaining thoraco-pulmonary compliance in the physiological range by preventing atelectasis, and reduced pulmonary inflammation [35]. In order to address the 2^nd^ hypothesis n = 8 animals in each group were randomly assigned to mechanical ventilation with 100% O_2_ (FiO_2_ 1.0), whereas the other mice were ventilated with air (FiO_2_ 0.21). Catheters were inserted into the jugular vein, the carotid artery, and the bladder. Anaesthesia was maintained with continuous i.v. ketamine (100–150 μg·g^-1^·h^-1^), midazolam (0.2–0.3 μg·g^-1^·h^-1^), and fentanyl (1–1.5 μg·g^-1^·h^-1^). Anaesthetic drugs were titrated to reach deep sedation and analgesia as documented by complete tolerance against noxious stimuli. To maintain mean arterial pressure > 50 mmHg animals received 12 μL·g^-1^· h^-1^ of hydroxyethyl starch in a balanced electrolyte solution (Tetraspan 6%, 130, 0.42, Braun Medical, Melsungen, Germany).

### Measurements

All animals were studied over four hours of mechanical ventilation. Systemic haemodynamics (heart rate, mean blood pressure) and body temperature were recorded hourly. The static thoraco-pulmonary compliance was measured hourly by incrementally increasing the airway pressure up to a maximum inspiratory pressure of 20 cm H_2_O and using the indwelling software of the respirator that allows automatic recording of the inspiratory and expiratory pressure-volume loop. Arterial blood samples were taken hourly for blood gases and pH. The respiratory rate was titrated to maintain arterial PCO_2_ at 30–40 mmHg, the PEEP level was titrated according to the arterial PO_2_: if the PaO_2_/FiO_2_ ratio was > 300 mmHg PEEP was decreased to 3 cmH_2_O, and if 100 mmHg < PaO_2_/FiO_2_ < 200 mmHg, it was increased to 8 cm H_2_O. At the end of the observation period, animals were killed through blood withdrawal via the carotid artery. Immediately thereafter the respiratory tubes were clamped at end-expiration, i.e. PEEP level, the thorax was opened, and the lungs were removed.

### Blood and lung tissue preparation

Whole blood was immediately spun, and plasma was stored at -80°C until analysis. The right lung was sampled, immediately frozen in liquid nitrogen and stored at -80°C for cytokine measurements, immunoblotting and EMSA. The left lung was fixed in formalin and paraffin-embedded for histology and immunohistochemistry.

### Cytokine concentrations

Plasma and lung tissue levels of the cytokines and chemokines tumour necrosis factor (TNF)-α, interleukin (IL)-1β, IL-6, IL-10, IL-18, keratinocyte chemoattractant (KC), and monocyte chemotactic protein-1 (MCP-1) were measured by a mouse multiplex cytokine kit (Bio-Plex Pro Cytokine Assay, Bio-Rad, Hercules, CA) in accordance to the manufacturer’s instructions [8,33,34]. In brief, the appropriate standards and samples were added to a filter plate. The samples were incubated with antibodies chemically attached to fluorescent-labelled micro beads. Thereafter, premixed detection antibodies were added to each well, and streptavidin-phycoerythrin was added. Beads were then re-suspended, and the cytokines reaction mixture was quantified using the Bio-Plex protein array reader. Data were automatically processed and analysed by Bio-Plex Manager Software 4.1 using the standard curve produced from recombinant cytokine standards. Levels below the detection limit of the assays were set to zero for statistical purposes. Due to technical problems, plasma cytokine concentrations are from n = 7 animals in the “CS” groups, while lung tissue levels are from n = 6 and n = 7 of the air-ventilated “non-CS” and “CS” mice, respectively.

### Cell extracts, EMSA, and immune-blots

Lung tissue was homogenized and lysed in lysing buffer. For cell extract preparation, cells were re-suspended, lysed on ice and centrifuged. The supernatant (protein extract) was stored at -80°C. For the assessment of the expression of haeme oxygenase-1 (HO-1), cleaved caspase-3 and HIF-1α, protein concentrations were determined, and equal total protein aliquots (20–60 μg) were separated by SDS-PAGE and transferred by Western blotting [8,33]. After blocking, the membranes were incubated with commercially available primary antibodies (anti-HO-1, Abcam, Cambridge, NY; anti-cleaved caspase 3, Cell Signaling, Danvers, MA; anti-HIF-1α, Thermo Fisher Scientific, Waltham, MA). The primary antibodies were detected using horseradish peroxidase-conjugated secondary antibodies (Cell Signaling, Danvers, MA or Santa Cruz, Dallas, TX). The membranes were subjected to chemo-luminescence using SuperSignal West Femto Maximum Sensitivity Substrate (Thermo Fisher Scientific). Exposed films were scanned, and intensity of immune-reactivity was measured using NIH Image J software (http://rsb.info.nih.gov/nih-image). Actin and vinculin (Santa Cruz Biotechnology, Inc., Santa Cruz, CA) served as the loading controls. All immune-blots were repeated twice.

Activation of the nuclear transcription factor κB (NF-κB) was determined using electrophoretic mobility shift assay (EMSA) as described recently [8,33,34]: cell extracts were incubated with Poly-doxy-inosinic-deoxy-cytidylic acid (poly-dI-dC) and ^32^P -labeled double stranded oligonucleotide containing the NF-кB (HIVкB-site) (5’-AGT TGA GGG GAC TTT CCC AGG C-3’, Biomers, Ulm). Complexes were separated in polyacrylamide gels and exposed to X-ray films. A phosphor-imager and image analysis software (AIDA Image Analyzer, Raytest, Straubenhardt) allowed quantifying the radioactively labeled NF-κB.

For comparison between individual gels, the intensity of each band was related to that of two loaded control animals which had not undergone surgical instrumentation and trauma. Therefore, the immune-blot and EMSA data are expressed as fold increase over the mean of the two control values using the mean value of the three individual gels for each experimental animal.

### Histology and immunohistochemistry

The left lung was formalin fixed and embedded in paraffin for histological and immunohistochemistry analysis. Adapting recently published scoring systems [36] the haematoxylin eosin-stained lung sections were analysed by two experienced pathologist (A.S., P.M.) blinded for the group assignment for alveolar collapse (i.e. dystelectasis or atelectasis), emphysematous over-distension, inflammatory cell (i.e. neutrophils, macrophages, and lymphocytes, respectively) infiltration, thickening of the alveolar membranes, protein debris in the airspaces, and alveolar oedema. These parameters were scored on a scale ranging from 0 (normal lung histology without pathological findings), 0.5 (minor histological injury), 1 (moderate and patchy histological injury), 1.5 (major histological injury < 25% of the lung involved), and 2 (major histological injury 25–50% of the lung involved).

Immunohistochemistry detection of nitrotyrosine formation and expression of the purinergic receptors 2X_7_ and 2X_4_ (P2XR_7_, P2XR_4_) was performed as follows: the paraffin sections were deparaffinized in xylene and graded ethanols, boiled twice in sodium citrate buffer for heat-induced epitope retrieval before being exposed to the primary antibody (anti-nitrotyrosine, Millipore, Schwalbach, Germany; anti-P2X_7_-Receptor and -P2X_4_-Receptor, Alomone Labs, Jerusalem, Israel,). Primary antibody detection was performed by a secondary antibody and visualized with a red chromogen (Alkaline Phosphatase-conjugated Goat-Anti-Rabbit IgG, Jackson Immuno Research,West Grove, USA). Slides were visualized using a Zeiss Axio Imager A1 microscope using a 10x objective (EC Plan-NEOFLUAR). Four distinct random 800 μm^2^ square regions were quantified for intensity of signal using the image analysis AxioVision 4.8 software (Zeiss, Jena, Germany). Therefore, results are presented as densitometric sum red [8,34].

### Statistical analysis

All data are presented as median (quartiles) unless otherwise stated. The sample sizes were based on our previous experience [8], for which a statistical power analysis using the Horovitz-index (PaO_2_/FiO_2_ ratio), thoraco-pulmonary compliance and lung tissue NF-κB activation as main criteria and based on two-sided testing, α = 0.05, power 80% and non-parametric analysis of variance had yielded a minimum of n = 8–10 for eight experimental groups. The scope of the present study was to assess the effects of CS-exposure prior to and hyperoxia immediately after *blunt chest trauma*. Therefore, in an attempt to reduce the number of animals, we did not study sham-operated mice and used n = 8 in each of the four experimental groups. After exclusion of normal distribution using the Kolmogorov-Smirnov-test, intergroup differences were analysed with a Kruskal-Wallis one way ANOVA on ranks, and a subsequent Dunn’s test for multiple comparisons using a two-tailed hypothesis testing. Differences were considered statistically significant when p < 0.05. All quantitative graphical presentations and statistical analyses were performed using the GraphPad Prism 5, version 5.04, software (GraphPad Software Inc., La Jolla, CA).

## Results

### Effects of cigarette smoke-exposure

Table 1 demonstrates that systemic haemodynamics, metabolism, and acid-base status did not differ between the four experimental groups. However, CS exposure prior to chest trauma was associated with a higher static thoraco-pulmonary compliance post trauma and lower PaO_2_ and Horovitz-index.

**Table 1:** Parameters of systemic hemodynamics, lung mechanics, pulmonary gas exchange, metabolism, and acid-base. Parameters of systemic hemodynamics, lung mechanics, pulmonary gas exchange, metabolism, and acid-base status in mice without (Non-CS) and with (CS) cigarette smoke exposure over three to four weeks prior to blunt chest trauma and after four hours mechanical ventilation with air (FiO_2_ 0.21) or 100% O_2_ (FiO_2_ 1.0) (n = 8 in each group). All data are median (quartiles).

	Non-CS FiO_2_ 0.21	CS FiO_2_ 0.21	Non-CS FiO_2_ 1.0	CS FiO_2_ 1.0	p-value K-W ANOVA
**Heart rate [min** ^**-1**^ **]**	340 (340;348)	355 (320;375)	395 (364;443)	322 (318;350)	0.089
**Mean arterial pressure [mmHg]**	54 (54;58)	56 (52;57)	57 (56;60)	58 (55;60)	0.326
**Glucose [mmol·L** ^**-1**^ **]**	5.7 (5.4;6.6)	4.5 (3.7;4.8) §	5.0 (4.4;6.4)	5.2 (4.8;5.8)	0.024
**Lactate [mmol·L** ^**-1**^ **]**	1.8 (1.1;2.0)	1.1 (0.9;1.1)	1.2 (0.8;1.3)	0.9(0.8;1.5)	0.199
**Minute ventilation [L·min** ^**-1**^ **]**	750 (745;810)	810 (768;828)	800 (760;830)	750 (748;758)	0.227
**Compliance [L·cm H**_**2**_ **O** ^**-1**^ **]**	79 (75;93)	97 § (82;106)	75 (65;85)	90 § (87;96)	0.028
**PaO**_**2**_ **[mmHg]**	102 (97;106)	78 § (75;82)	379 $ (318;395)	370 $ (329;424)	< 0.001
**PEEP [cm H**_**2**_ **O]**	3.0 (3.0;3.0)	3.0 (3.0;3.5)	3.0 (3.0;3.0)	3.0 (3.0;3.5)	0.272
**PaO**_**2**_ **/FiO**_**2**_ **ratio [mmHg]**	491 (460;505)	368 § (358;391)	379 $ (318;395)	370 (329;424)	0.007
**PaCO**_**2**_ **[mmHg]**	35 (32;38)	32 (32;35)	37 (29;37)	37 (34;43)	0.460
**Arterial pH**	7.32 (7.30;7.34)	7.30 (7.23;7.33)	7.32 (7.31;7.35)	7.30 (7.28;7.34)	0.565
**Arterial base excess [mmol·L** ^**-1**^ **]**	-7.4 (-8.5;-6.9)	-8.1 (-9.7;-6.8)	-6.8 (-8.0;-6.5)	-7.0 (-10.0;-5.4)	0.617

^§^ depicts p < 0.05 vs. the corresponding Non-CS group,

^$^ depicts p < 0.05 vs. the corresponding air group (K-W ANOVA Kruskall-Wallis analysis of variance on ranks with post-hoc Dunn’s test for multiple comparisons).

Table 2 shows that CS exposure prior to trauma increased post-traumatic lung tissue IL-18 and blood MCP-1 concentrations, the latter, however, did not reach statistical significance (p < 0.1 each during air and O_2_ ventilation).

**Table 2:** Plasma and lung tissue cytokine and chemokine concentrations. Plasma (in pg·mL^-1^) and lung tissue (in pg·mg_protein_^-1^) cytokine and chemokine concentrations at the end of the four-hours observation period (n = 8 in each group) obtained from mice without (Non-CS) and with (CS) cigarette smoke exposure over 3–4 weeks prior to blunt chest trauma and mechanical ventilation with air (FiO_2_ 0.21) or 100% O_2_ (FiO_2_ 1.0). All data are median (quartiles).

	Non-CS FiO_2_ 0.21	CS FiO_2_ 0.21	Non-CS FiO_2_ 1.0	CS FiO_2_ 1.0	p-value K-W ANOVA
**IL-1β [pg·mL** ^**-1**^ **]**	32 (25;36)	37 (22;49)	23 (13;29)	13 (9;19)	0.215
**IL-1β [pg·mg**_**protein**_^**-1**^ **]**	309 (209;457)	531 (204;631)	223 (118;332)	285 (245;325)	0.413
**IL-6 [pg·mL** ^**-1**^ **]**	212 (71;475)	511 (310;863)	123 (93;275)	177 (122;434)	0.304
**IL-6 [pg·mg**_**protein**_^**-1**^ **]**	5 (5;20)	9 (8;11)	11 (8;18)	5 (4;7)	0.116
**IL-10 [pg·mL** ^**-1**^ **]**	99 (72;171)	153 (92;255)	85 (53;126)	95 (80;177)	0.467
**IL-10 [pg·mg**_**protein**_^**-1**^ **]**	47 (43;53)	38 (34;40)	42 (39;45)	48 (45;51)	0.153
**KC [pg·mL** ^**-1**^ **]**	275 (146;421)	265 (156;644)	115 (97;136)	223 (212;324)	0.369
**KC [pg·mg**_**protein**_^**-1**^ **]**	570 (313;833)	606 (469;671)	320 (224;497)	450 (391;586)	0.204
**MCP-1 [pg·mL** ^**-1**^ **]**	772 (528;2517)	1395 (889;7011)	265 (208;779)	3060 (1859;3389)	0.099
**MCP-1 [pg·mg**_**protein**_^**-1**^ **]**	141 (135;156)	137 (123;166)	136 (114;150)	129 (121;135)	0.622
**TNF-α [pg·mL** ^**-1**^ **]**	90 (84;104)	111 (82;137)	86 (61;93)	93 (86;108)	0.456
**TNF-α [pg·mg**_**protein**_^**-1**^ **]**	113 (75;133)	113 (102;121)	122 (111;145)	113 (88;144)	0.707
**IL-18 [pg·mL** ^**-1**^ **]**	234 (165;464)	189 (169;217)	179 (147;220)	148 (105;208)	0.429
**IL-18 [pg·mg**_**protein**_^**-1**^ **]**	603 (533;673)	973 (795;1045) §	563 (440;600)	882 (629;1088) §	0.019

^§^ depicts p < 0.05 vs. the corresponding Non-CS group,

^$^ depicts p < 0.05 vs. the corresponding air group (K-W ANOVA Kruskall-Wallis analysis of variance on ranks with post-hoc Dunn’s test for multiple comparisons).

Table 3 demonstrates that the CS exposure-induced alterations in pulmonary gas exchange and lung cytokine concentrations coincided with a two- to four-fold higher tissue infiltration of neutrophils, smokers’ macrophages, and lymphocytes, as well as with more pronounced alveolar wall thickening.

**Table 3:** Parameters of lung histopathology. Parameters of lung histopathology at the end of the four-hours observation period (n = 8 in each group) obtained from mice without (Non-CS) and with (CS) cigarette smoke exposure over 3–4 weeks prior to blunt chest trauma and mechanical ventilation with air (FiO_2_ 0.21) or 100% O_2_ (FiO_2_ 1.0) (n = 8 in each group). Parameters were scored as 0 (normal lung histology without pathological findings), 0.5 (minor histological injury), 1 (moderate and patchy histological injury), 1.5 (major histological injury < 25% of the lung involved), and 2 (major histological injury 25–50% of the lung involved). All data are median (quartiles).

	Non-CS FiO_2_ 0.21	CS FiO_2_ 0.21	Non-CS FiO_2_ 1.0	CS FiO_2_ 1.0	p-value K-W ANOVA
**Dystelectasis/atelectasis**	1.0 (0.5;1.0)	0.5 (0.5;1.0)	1.0 (0.5;1.0)	0.5 (0.5;0.6)	0.683
**Emphysema**	1.5 (1.5,2.0)	1.5 (1.0;1.5)	1.3 (1.0;1.5)	1.1 § (1.0;1.3)	0.011
**Alveolar membrane thickening**	1.0 (1.0;1.0)	1.5 § (1.5;1.8)	1.0 (1.0;1.5)	1.5 § (1.4;1.6)	0.047
**Lymphocytes**	1.0 (1.0;1.0)	2.0 § (1.8;2.3)	1.5 (1.5;2.0)	2.0 § (1.9;2.5)	< 0.001
**Neutrophils**	0.5 (0.5;0.8)	2.0 § (2.0;2.5)	0.8 (0.5;1.0)	1.5 § (1.5,2.0)	< 0.001
**Macrophages**	3.5 (3.3;3.8)	12.0 § (7.0;13.5)	3.5 (3.0;5.6)	14.0 § (11.5;15.8)	< 0.001
**Protein debris in the airspaces**	1.0 (0.5;1.0)	0.5 (0.5;0.8)	1.0 (1.0;1.0)	0.8 (0.5;1.0)	0.074
**Alveolar edema**	0.5 (0.3;0.5)	0.5 (0.5;1.0)	1.0 (0.9;1.1)	1.5 § (0.9;2.0)	0.022

^§^ depicts p < 0.05 vs. the corresponding Non-CS group,

^$^ depicts p < 0.05 vs. the corresponding air group (K-W ANOVA Kruskall-Wallis analysis of variance on ranks with post-hoc Dunn’s test for multiple comparisons).

**Fig 1:**
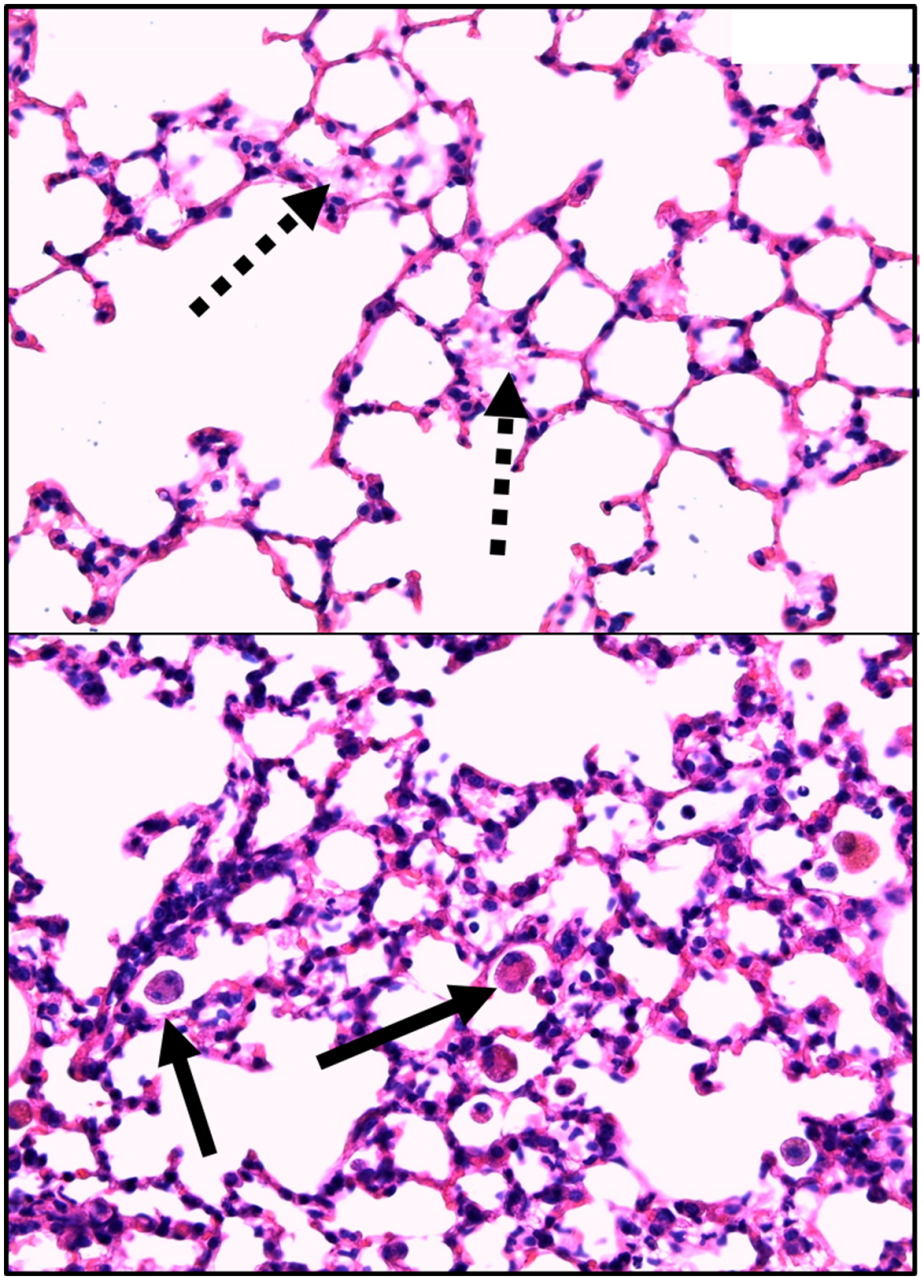
Typical examples of the histopathological items scored. *Upper panel*: Lung region with little histopathological abnormalities, i.e. no dystelectasis/ atelectasis, normal thickness of alveolar membranes, and little lymphocyte immigration. Dotted arrows show some degree of protein debris in the airspaces. *Lower panel*: Lung region with major histological injury (alveolar membrane thickening and lymphocyte immigration grade 1.5 each). Solid arrows show alveolar smokers’ macrophages within the airways.

**Fig 2:**
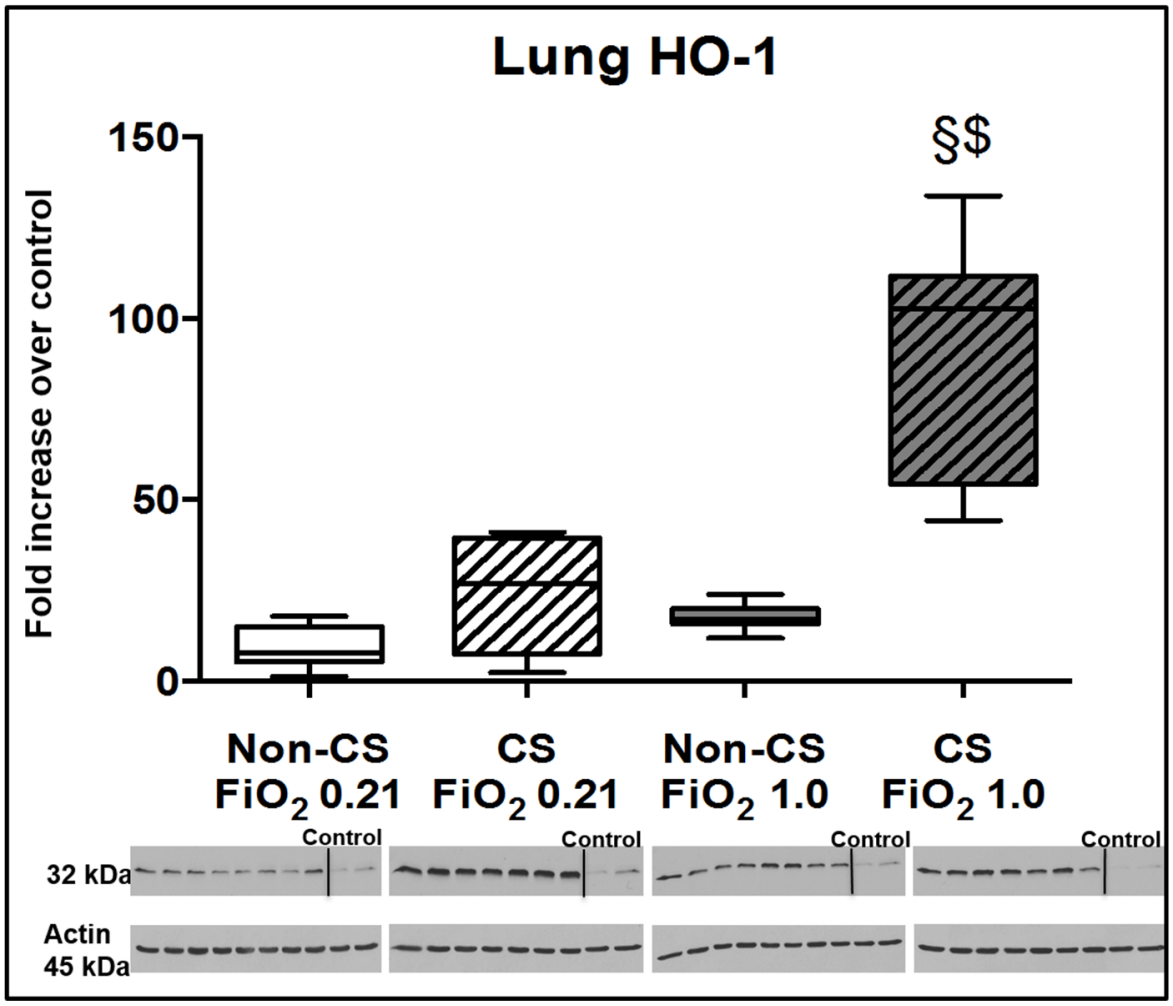
Results of the immune blotting for HO-1. Original western blots and quantitative analysis of lung tissue expression of HO-1 from mice without (open boxplots; n = 8 each) and with (hatched boxplots; n = 7 each) cigarette smoke exposure prior to blunt chest trauma and mechanically ventilated with air (white boxplots) and 100% O_2_ (grey boxplots) together with two blots each (right part of blot panel) from control animals that did not undergo cigarette smoke exposure, anaesthesia, chest trauma, and surgery. All data are median (quartiles, range) as fold increase over values from control animals; § p < 0.05 vs. corresponding cigarette smoke exposure group, $ p < 0.05 vs. corresponding air ventilation group (Kruskall-Wallis analysis of variance on ranks with post-hoc Dunn’s test for multiple comparisons).

**Fig 3:**
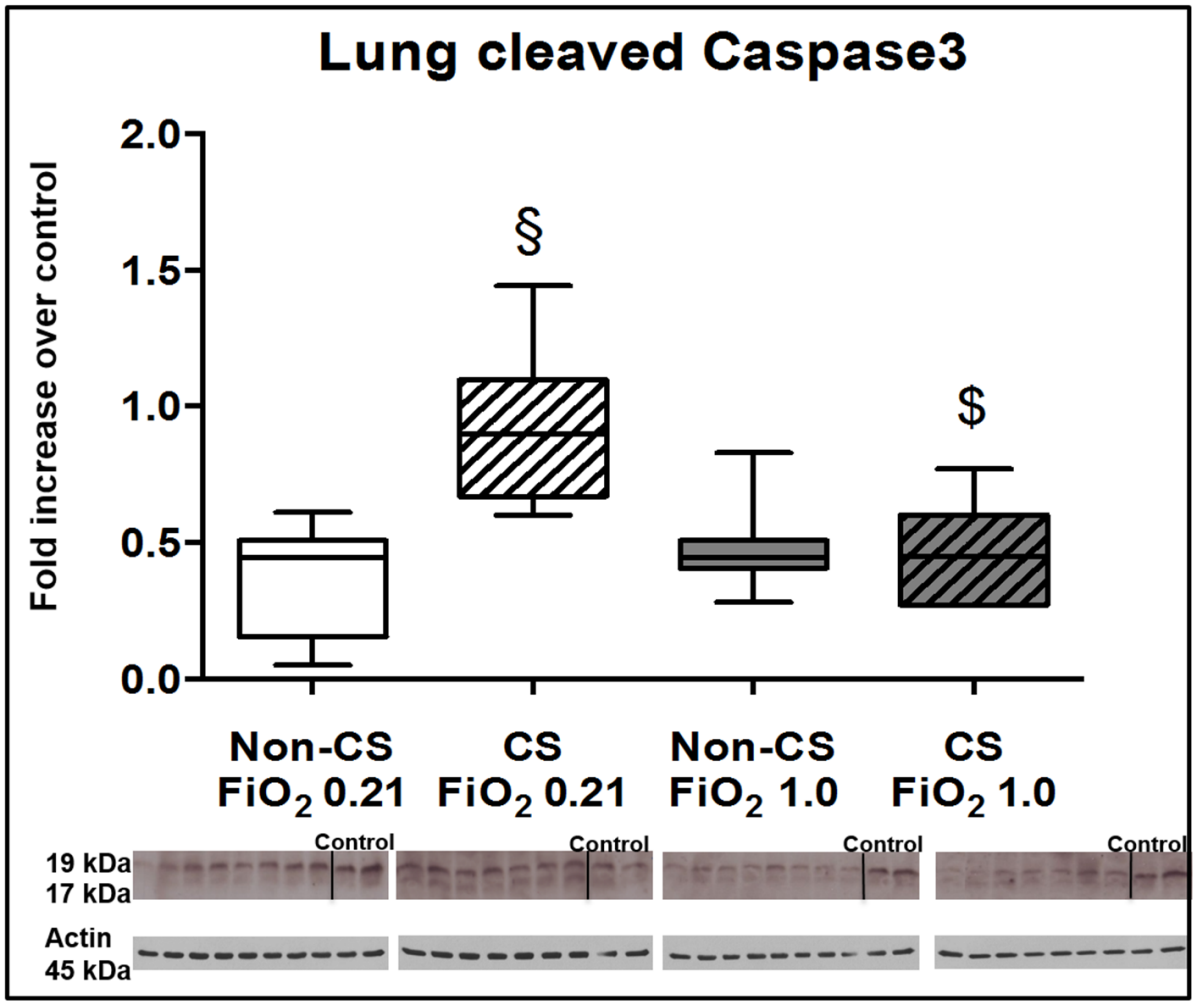
Results of the immune blotting for activated caspase-3. Original western blots and quantitative analysis of lung tissue expression of activated caspase-3 from mice without (open boxplots; n = 8 each) and with (hatched boxplots; n = 7 each) cigarette smoke exposure prior to blunt chest trauma and mechanically ventilated with air (white boxplots) and 100% O_2_ (grey boxplots) together with two blots each (right part of blot panel) from control animals that did not undergo cigarette smoke exposure, anaesthesia, chest trauma, and surgery. All data are median (quartiles, range) as fold increase over values from control animals; § p < 0.05 vs. corresponding cigarette smoke exposure group, $ p < 0.05 vs. corresponding air ventilation group (Kruskall-Wallis analysis of variance on ranks with post-hoc Dunn’s test for multiple comparisons).

**Fig 4:**
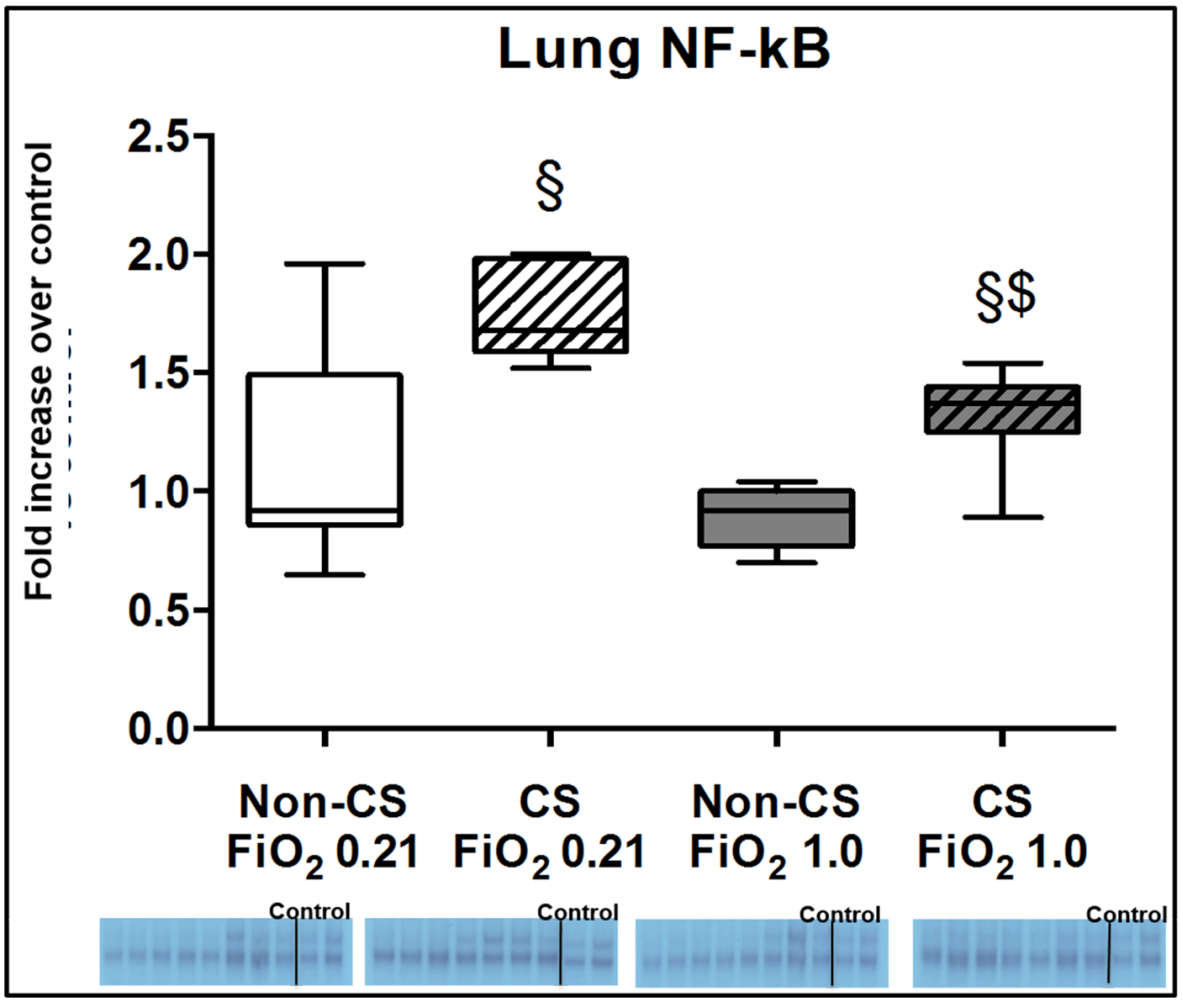
Results of the EMSA for NF-κ activation. Original gel shifts and quantitative analysis of lung tissue expression of NF-κB activation from mice without (open boxplots; n = 8 each) and with (hatched boxplots; n = 7 each) cigarette smoke exposure prior to blunt chest trauma and mechanically ventilated with air (white boxplots) and 100% O_2_ (grey boxplots) together with two blots each (right part of blot panel) from control animals that did not undergo cigarette smoke exposure, anaesthesia, chest trauma, and surgery. All data are median (quartiles, range) as fold increase over values from control animals; § p < 0.05 vs. corresponding cigarette smoke exposure group, $ p < 0.05 vs. corresponding air ventilation group (Kruskall-Wallis analysis of variance on ranks with post-hoc Dunn’s test for multiple comparisons).

**Fig 5:**
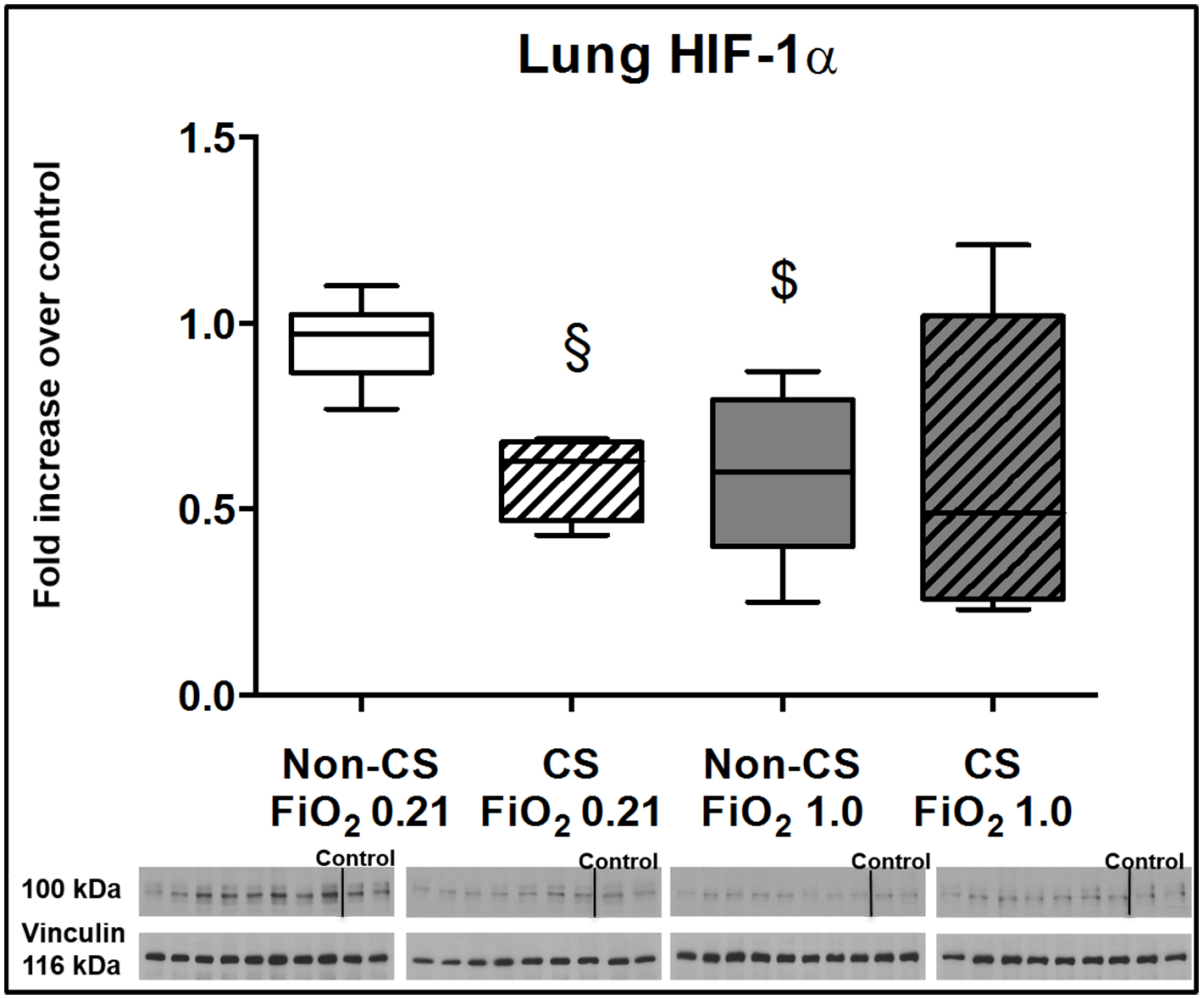
Results of the immune blotting for HIF-1α. Original western blots and quantitative analysis of lung tissue expression of HIF-1α expression from mice without (open boxplots; n = 8 each) and with (hatched boxplots; n = 7 each) cigarette smoke exposure prior to blunt chest trauma and mechanically ventilated with air (white boxplots) and 100% O_2_ (grey boxplots) together with two blots each (right part of blot panel) from control animals that did not undergo cigarette smoke exposure, anaesthesia, chest trauma, and surgery. All data are median (quartiles, range) as fold increase over values from control animals; § p < 0.05 vs. corresponding cigarette smoke exposure group, $ p < 0.05 vs. corresponding air ventilation group (Kruskall-Wallis analysis of variance on ranks with post-hoc Dunn’s test for multiple comparisons).

**Fig 6:**
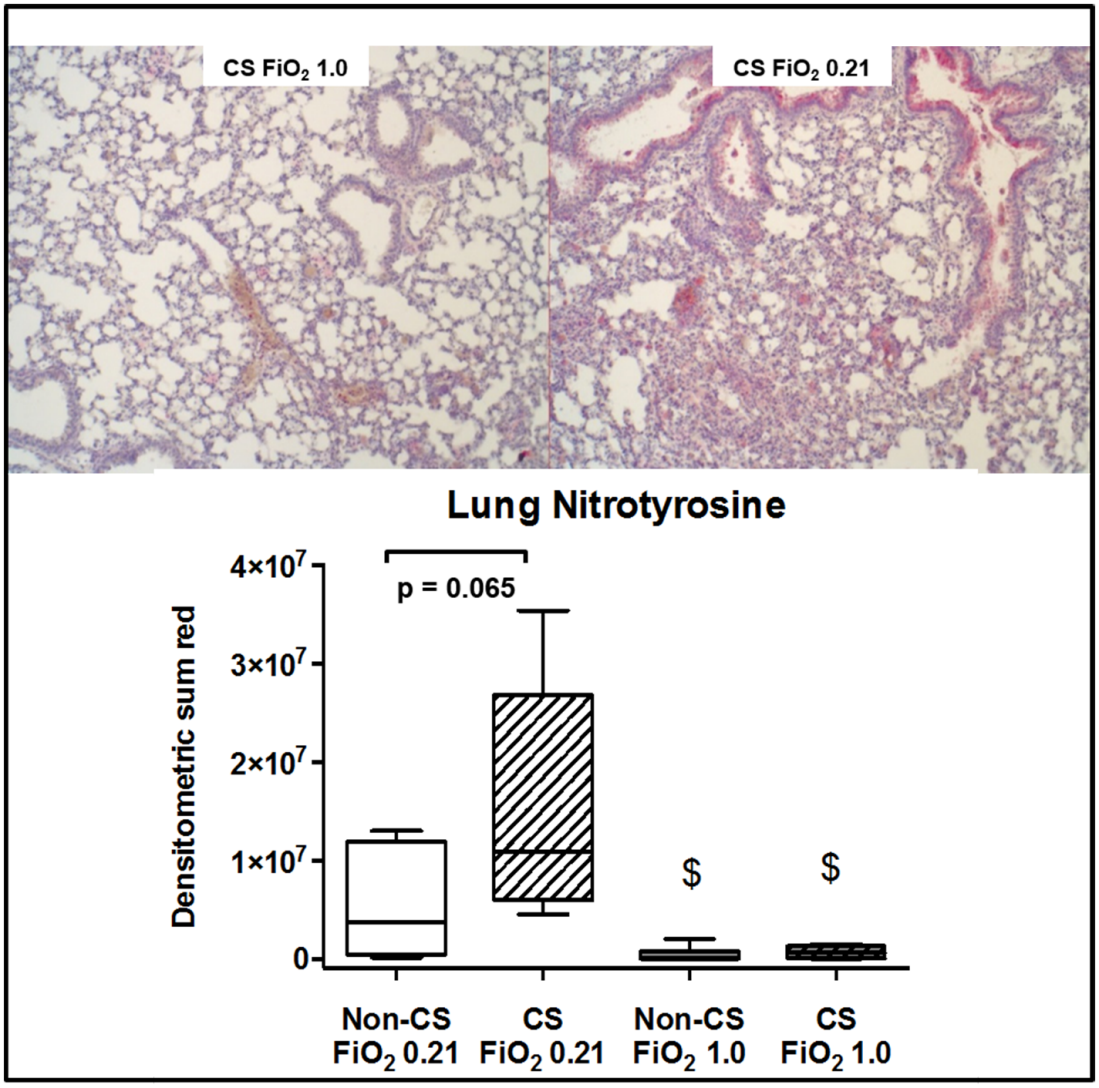
Results of the immunohistochemistry for nitrotyrosine. Typical examples (upper panel) and quantitative analysis (lower panel) of immunohistochemistry for lung tissue nitrotyrosine formation from mice without (dotted boxplots) and with (hatched boxplots) cigarette smoke exposure prior to blunt chest trauma and mechanically ventilated with air (white boxplots) and 100% O_2_ (grey boxplots) (n = 8 in each group). All data median (quartiles, range), § p < 0.05 vs. corresponding cigarette smoke exposure group, $ p < 0.05 vs. corresponding air ventilation group (Kruskall-Wallis analysis of variance on ranks with post-hoc Dunn’s test for multiple comparisons).

**Fig 7:**
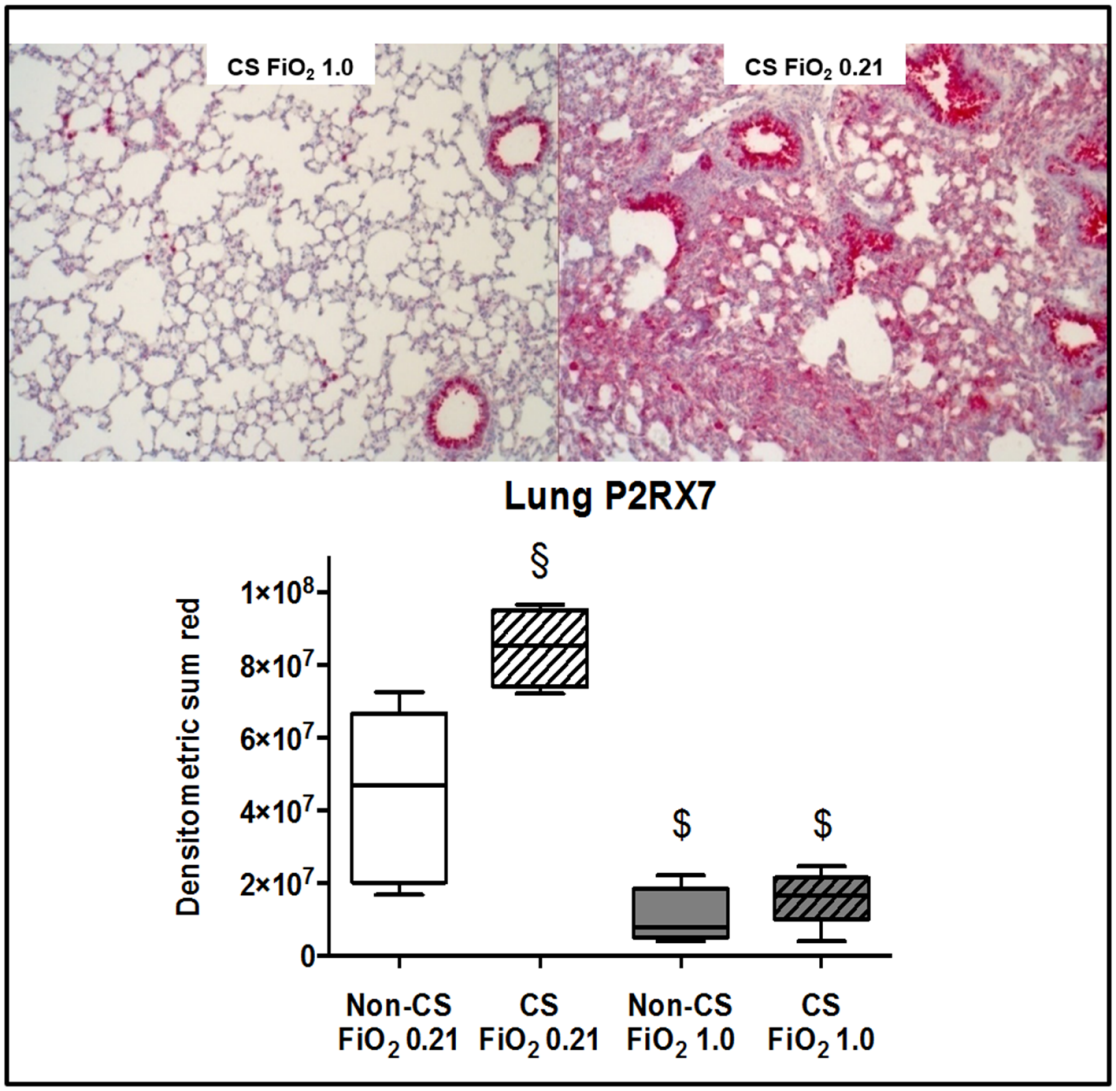
Results of the immunohistochemistry for P2X_7_. Typical examples (upper panel) and quantitative analysis (lower panel) of immunohistochemistry for lung tissue expression of the purinergic receptor P2X_7_ from mice without (dotted boxplots) and with (hatched boxplots) cigarette smoke exposure prior to blunt chest trauma and mechanically ventilated with air (white boxplots) and 100% O_2_ (grey boxplots) (n = 8 in each group). All data median (quartiles, range), § p < 0.05 vs. corresponding cigarette smoke exposure group, $ p < 0.05 vs. corresponding air ventilation group (Kruskall-Wallis analysis of variance on ranks with post-hoc Dunn’s test for multiple comparisons).

**Fig 8:**
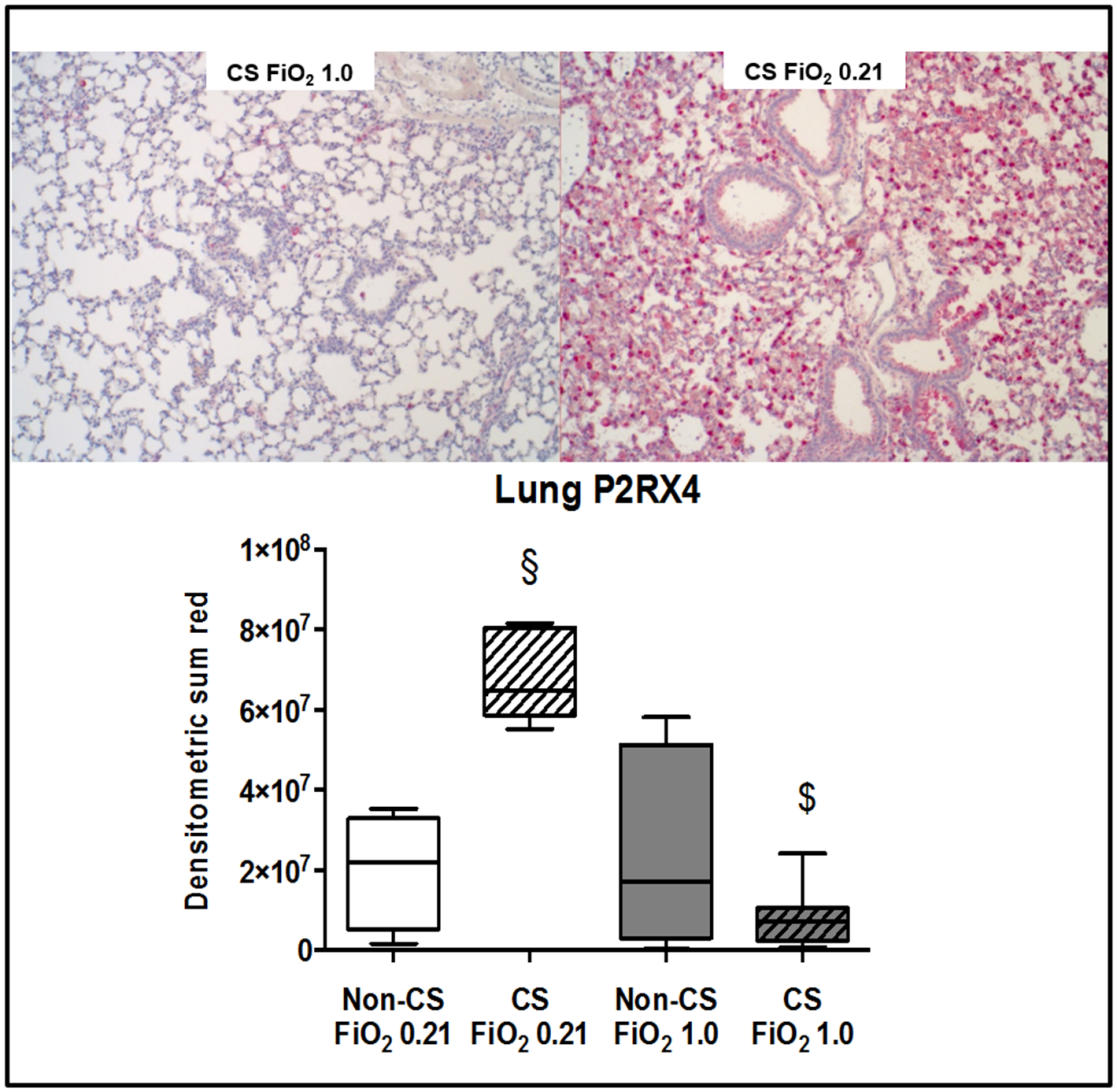
Results of the immunohistochemistry for P2X_4_. Typical examples (upper panel) and quantitative analysis (lower panel) of immunohistochemistry for lung tissue expression of the purinergic receptor P2X_4_ from mice without (dotted boxplots) and with (hatched boxplots) cigarette smoke exposure prior to blunt chest trauma and mechanically ventilated with air (white boxplots) and 100% O_2_ (grey boxplots) (n = 8 in each group). All data median (quartiles, range), § p < 0.05 vs. corresponding cigarette smoke exposure group, $ p < 0.05 vs. corresponding air ventilation group (Kruskall-Wallis analysis of variance on ranks with post-hoc Dunn’s test for multiple comparisons).

Typical examples of the histological items are shown in Fig 1. Whereas CS exposure did not affect post-traumatic lung tissue HO-1 expression (Fig 2), it markedly increased activated caspase-3 (Fig 3) expression and NF-κB activation (Fig 4), and reduced HIF-1α formation (Fig 5). CS exposure was also associated with increased tissue nitrotyrosine formation (Fig 6), P2XR_7_ and P2XR_4_ expression (Figs 7 and 8).

### Effects of hyperoxia

Tables 1 and 2 demonstrate that lung-protective mechanical ventilation at FiO_2_ 1.0 did not further affect lung mechanics, gas exchange or pulmonary and systemic cytokine and chemokine concentrations. Table 3 shows that ventilation with 100% O_2_ did not affect lung histology except for some patchy alveolar edema in CS exposed mice, however, without any deleterious effect on organ function (see Table 1). While ventilation with 100% O_2_ markedly increased lung tissue HO-1 expression (Fig 2) in CS exposed mice, it did not further affect activated caspase-3 (Fig 3) and HIF-1α expression (Fig 4), or NF-κB activation (Fig 5), and even attenuated nitrotyrosine formation (Fig 6), and P2XR_7_, and P2XR_4_ expression (Figs 7 and 8).

## Discussion

This study investigated the effect of short-term hyperoxia on post-traumatic pulmonary and systemic inflammation and lung injury in mice that had undergone CS exposure prior to blunt chest trauma. The major findings were that i) CS exposure prior to trauma aggravated trauma-induced lung dysfunction and organ injury due to more pronounced pulmonary inflammation and nitrosative stress, ultimately resulting in enhanced apoptosis and tissue damage, and that ii) lung-protective mechanical ventilation with 100% O_2_ partially attenuated this effect.

### Effects of cigarette smoke-exposure on the acute trauma response

Smoking is directly related to the development of ALI after severe blunt trauma [2]. Our findings well agree with this observation: mice that had undergone CS exposure prior to trauma showed impaired pulmonary gas exchange and more pronounced lung histological damage. Despite the comparable histological evidence of emphysematous lung over-distension, the higher static thoraco-pulmonary compliance in the CS exposed mice further supports this notion: CS abuse is the most important etiological factor for COPD [37], which in turn, is associated with a hyperinflation-induced increase of the static compliance [38], even when total lung capacity is still normal [39].

Murine models of CS-induced COPD are characterised by pulmonary inflammation and mediator release: after four days of CS-exposure, animals presented with a several-fold increase of neutrophil accumulation and pro-inflammatory cytokine and chemokine concentrations in the bronchoalveolar lavage (BAL) fluid [18,40]. In addition, CS exposure caused a two- to three-fold increase of markers of oxidative and nitrosative stress in the BAL fluid [41]. This effect resulted in enhanced apoptosis as documented by increased expression of the activated caspase-3 [41,42]. Finally, CS exposure increased ventilator-induced lung injury due to aggravated tissue inflammation and apoptosis in alveolear type II cells [4]. Our findings well agree with these observations: despite the 1-week recovery period aiming to avoid any procedure-related stress, CS-exposed mice presented with higher NF-κB activation, nitrotyrosine formation, and tissue IL-18 concentrations. The latter finding is of particular interest in the context of the CS exposure-related higher expression of the P2XR_7_ and P2XR_4_: inflammasome-regulated production of IL-1β and IL-18 has been referred to as a key mechanism of injurious ventilation- [43] and hyperoxia-induced [44] ALI *via* P2XR_7_ and subsequent inflammasome activation and IL-1β secretion: Genetic P2XR_7_ deletion reduced the endotoxin-induced release of IL-1β and thereby attenuated the impairment of lung mechanics and the histological organ damage *in vivo* [45], and P2XR_7_ activation aggravated the endotoxin-related vascular hypo-reactivity *in vitro* [46]. Co-expression of the P2XR_4_ enhanced the P2XR_7_-related inflammatory response [47], and up-regulation of P2XR_4_ was referred to compensate for P2XR_7_ depletion [48]. Finally, in addition to their role for the development of ALI, P2XR_7_ and P2XR_4_ activation was shown to assume major importance for CS-related lung injury: CS exposure caused up-regulation of both the P2XR_4_ and P2XR_7_ [49], and either pharmacological blockade or genetic deletion of the P2XR_7_ attenuated the pulmonary IL-1β and IL-18 accumulation after CS exposure [50]. Consequently, our experiments support P2XR_4_ and P2XR_7_ as potential therapeutic targets both in ALI [51] and CS-induced COPD [52,53].

Pre-traumatic CS exposure reduced lung tissue HIF-1α expression after chest trauma. At first glance, this finding is in contrast to data from both CS exposed animals [54] and patients with COPD [55,56]. The increased nitrosative stress in CS exposed mice may have assumed importance in this context: under normoxic conditions, hyper-inflammation-induced nitric oxide (NO) release impairs HIF-1α degradation due to inhibition of prolyl-hydroxylases (PHD) [57]. However, excess NO formation under hypoxic conditions may also reduce HIF-1α accumulation because of an NO-mediated feedback with expression of PHD and/or O_2_-redistribution to PHD resulting from NO-induced inhibition of mitochondrial respiration [58].

### Effects of hyperoxia on the acute trauma response

In mice without pre-traumatic CS exposure, pure O_2_ ventilation was associated with a significantly lower PaO_2_/FiO_2_ ratio than in the corresponding air-ventilated animals. This effect was most likely due to O_2_ breathing-induced instability of lung regions with low ventilation/perfusion-ratios [59] rather than due to O_2_ toxicity: O_2_ ventilation i) did not affect gas exchange or lung mechanics in mice after CS exposure, and ii) was not associated with any biochemical or morphological sign of aggravated pulmonary inflammation. Equivocal data are available whether short-term mechanical ventilation with 100% O_2_ induces a pulmonary and systemic inflammatory response: in rats, 120 minutes of pure O_2_ ventilation caused a marked increase in extravascular lung water and the BAL fluid neutrophil, chemokine and cytokine content, but this effect required the use of injurious tidal volumes (20 mL·kg^-1^). In contrast, tidal volumes similar to our study (7 mL·kg^-1^) had no effect [60]. In rabbits, four hours of mechanical ventilation with 100% O_2_ significantly increased BAL polymorphonuclear leukocytes and MCP-1 concentrations, associated with increased alveolar-capillary permeability. Again, this effect was only present with high (25 mL·kg^-1^) tidal volumes, while clinically more relevant (10 mL·kg^-1^) tidal volumes had no effects [61]. Finally, in mice incrementally increasing the FiO_2_ during 120 minutes of mechanical ventilation (tidal volume 7–8 mL·kg^-1^) did not alter airway resistance, tissue elastance nor BAL fluid concentrations of chemokines and cytokines [62]. Scarce data are only available on the effects of non-injurious mechanical ventilation with O_2_ in the presence of sepsis- or trauma-induced ALI: low-tidal volume (7 mL·kg^-1^) mechanical ventilation with 100% O_2_ caused more pronounced pulmonary and systemic inflammation than spontaneous air breathing, but the effects of hyperoxia *per se* remain open, since mechanically ventilation with lower FiO_2_ values was not studied [63]. In swine with fecal peritonitis-induced septic shock, lung-protective, pure O_2_ mechanical ventilation up to 24 hours did not cause any aggravation of pulmonary or systemic inflammation or histological damage [29,30].

Hyperoxia may have attenuated pulmonary and systemic inflammation by compensating regional pulmonary hypoxia as well as tissue hypoxia resulting from chest trauma-induced hypoxemia: hypoxic hypoxia (FiO_2_ 0.1) of incremental duration caused a time-dependent increase of the BAL fluid neutrophil count and albumin content reflecting alveolar-capillary leakage [64], and further aggravated endotoxin-induced ALI [65]. MCP-1 is a crucial mediator of this inflammatory response [66], and in our experiments, blood MCP-1 levels were three times lower in pure O_2_-ventilated mice without CS exposure prior to trauma (772 (528;2517) vs. 265 (208;779) pg·mL^-1^, p = 0.08). Pure O_2_ ventilation was also associated with reduced lung tissue nitrotyrosine formation, no matter whether mice had been exposed to CS or not. This finding well agrees with previous findings on the effects of short-term exposure to hyperoxia on oxidative and nitrosative stress: two or three hours of intermittent O_2_ breathing after zymosan injection increased the activity of tissue antioxidant enzymes [31], and long-term exposure to FiO_2_ 0.8 attenuated lung nitrotyrosine formation with intra-tracheal carrageenan-induced pneumonitis [67]. Hence, our findings suggest that under acute stress, short-term hyperoxia can counteract the nitrosative stress associated with CS exposure-induced COPD. Hyperoxia attenuated the P2XR_7_ expression both in mice with and without CS exposure prior to chest trauma, whereas it reduced P2XR_4_ expression only in animals with CS exposure. Hence, short-term hyperoxia may be of particular interest in acute stress under conditions of chronic hypox(aem)ia: hypoxic hypoxaemia induced by exposure to simulated altitude not only increased lung tissue P2XR_4_ mRNA, but also in the right ventricle, which went alongside with pulmonary artery hypertension and consecutive right ventricular hypertrophy [68].

Albeit we lack a direct proof, it is tempting to speculate that the hyperoxia-related attenuation of pulmonary and systemic inflammation was due to down-regulation of HIF-1α expression: Both genetic deletion [69,70] and pharmacological blockade [71] of HIF-1α attenuated the local and systemic inflammatory response and thereby reduced the severity of ALI after remote [69,70] or direct [71] organ damage. Finally, the neuro-protective effect of exposure to both hyper- and normobaric O_2_ was associated with a two to three-fold reduction of HIF-1α expression [72].

### Limitations of the study

It could be argued that despite the marked differences in the inflammatory response, the CS-related effects on lung mechanics, gas exchange, and histology lack physiological significance: overall they were moderate, and the Horovitz-index always remained above 300 mmHg, i.e. above the definition threshold of ALI. Of note, we [8,51] and others [73] found a similar dissociation between significantly increased levels of inflammatory biomarkers in the lung and lacking effects on lung mechanics and gas exchange in mice after chest trauma [8,51] and poly-microbial sepsis [73]. Only few studies reported Horovitz-indices compatible with the definition of ALI (< 300 mmHg) in mechanically ventilated mice: animals were either ventilated with injurious tidal volumes (12–40 mL·kg^-1^) [74–78], or 24 hours after injection of endotoxin, i.e. in the presence of prolonged, severe ALI [79]. Moreover, we used pressure-controlled, lung-protective ventilation that also comprised an initial lung recruitment manoeuver. Consequently, any further damage beyond the effect of lung contusion *per se* or due to injurious mechanical ventilation was avoided. In fact, neither pulmonary mechanics nor gas exchange deteriorated during the 4-hour observation period. Our approach is in contrast to previous experiments, which explicitly studied the effect of CS on tissue inflammation and apoptosis related to ventilator-induced lung injury [4]. However, in that study, data on lung mechanics, gas exchange or histological changes were not reported at all.

Clearly, the short duration of the mechanical ventilation precludes any conclusion on the long-term effects. However, a recent review article showed that only two studies described mechanical ventilation in mice of up to eight hours, while in the other reports only four to six hours of mechanical ventilation were used [80]. Finally, we cannot exclude that correcting trauma-related hypoxaemia using an FiO_2_ of 0.3–0.4 rather than air ventilation in the control group may have produced similar or even better results when compared to the hyperoxia group.

## Conclusion

In a murine model of COPD induced by CS exposure, pulmonary and systemic inflammation as well as nitrosative stress were aggravated after blunt chest trauma, which ultimately resulted in increased severity of post-traumatic organ dysfunction and injury as documented by impaired gas exchange and more pronounced histological damage. Overall, short-term, lung-protective mechanical ventilation with 100% O_2_ did not have a major therapeutic effect despite attenuation of nitrosative stress. The latter was possibly due to correction of regional alveolar hypoxia and/or consecutive hypoxemia, resulting in HIF-1α down-regulation.
